# Supplementary figures and images for: Synthesis of novel technetium-99m tricarbonyl-HBED-CC complexes and structural prediction in solution by density functional theory calculation
Source: R Soc Open Sci. 2019 Nov 27;6(11):191247. doi: 10.1098/rsos.191247 (PMC6894603; doi:10.1098/rsos.191247)

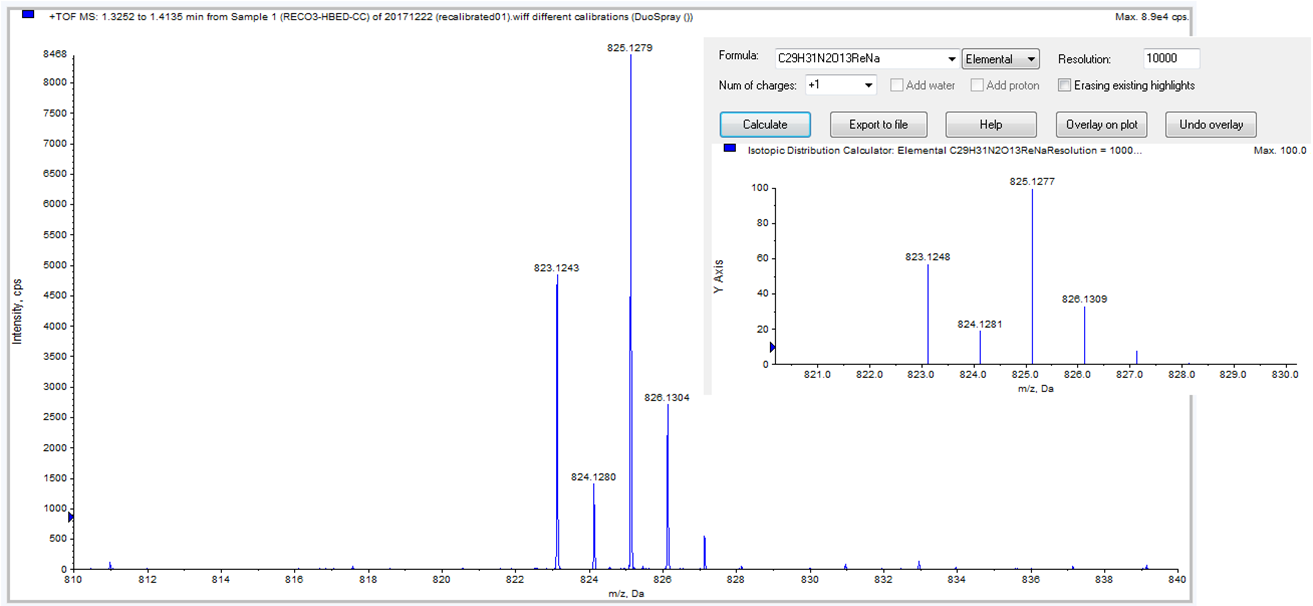


Figure S1 HRMS of Re(CO)3**L1**

Supplement: Figure S1 HRMS of Re(CO)3L1 [file rsos191247supp1.docx]

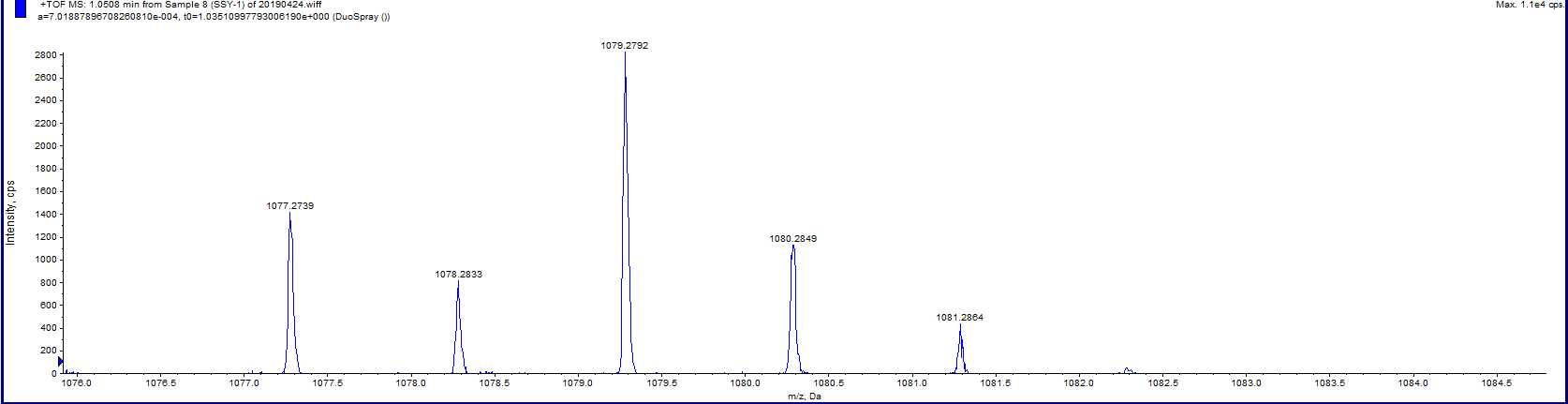


Figure S2 HRMS of Re(CO)3**L2**

Supplement: Figure S2 HRMS of Re(CO)3L2 [file rsos191247supp2.docx]

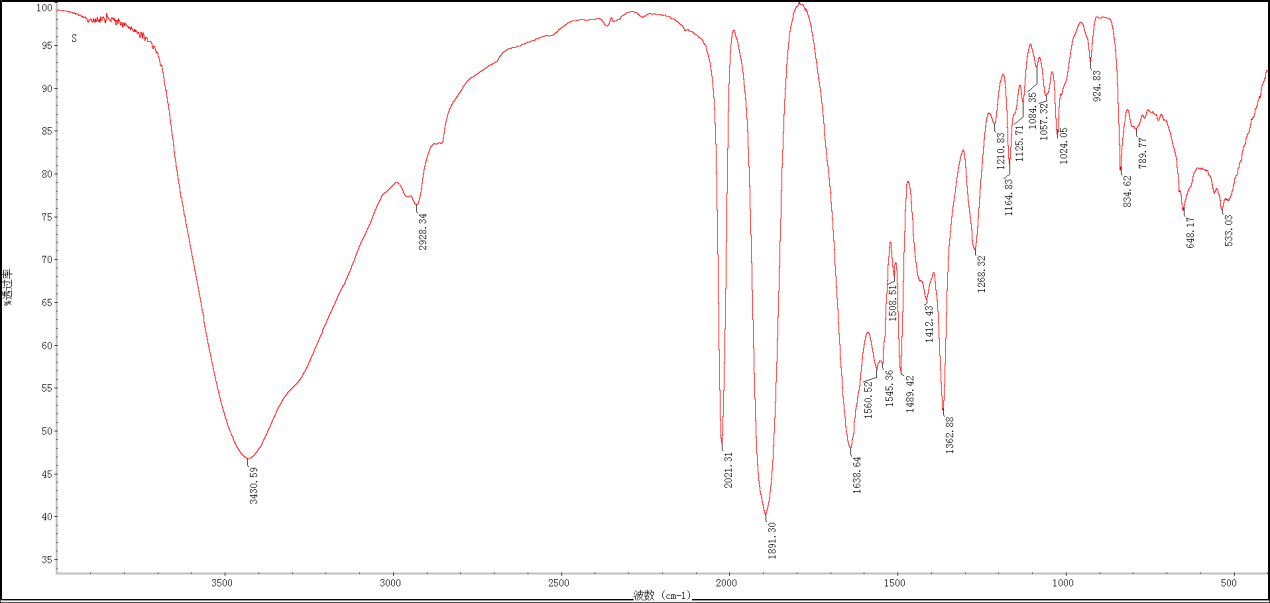


Figure S3 IR spectroscopy of Re(CO)3**L2**

Supplement: Figure S3 IR of Re(CO)3L2 [file rsos191247supp3.docx]

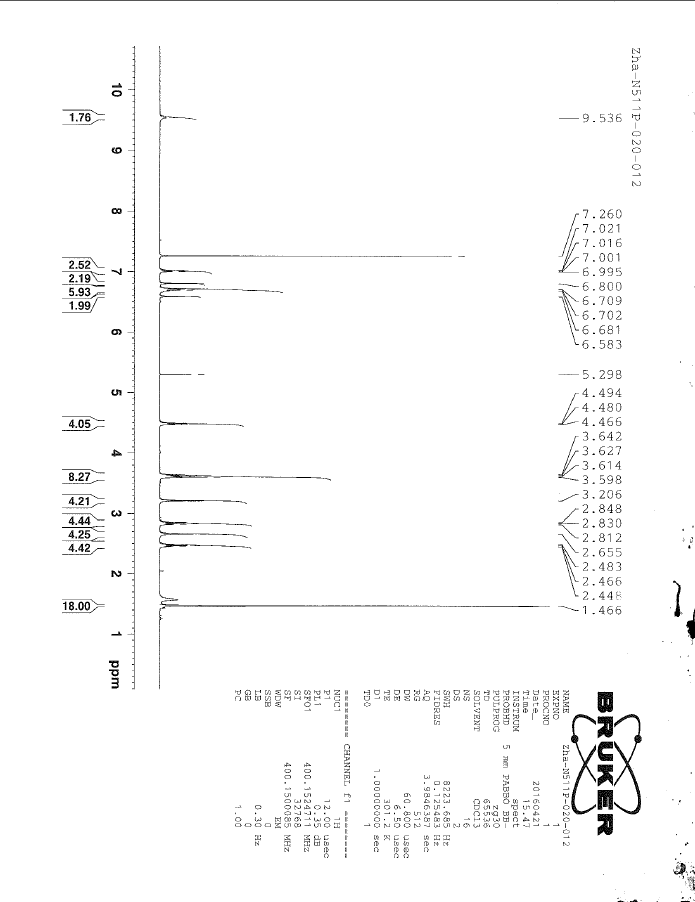


Figure S4 1H NMR of compound **8**

Supplement: Figure S4 1H NMR of compound 8 [file rsos191247supp4.docx]

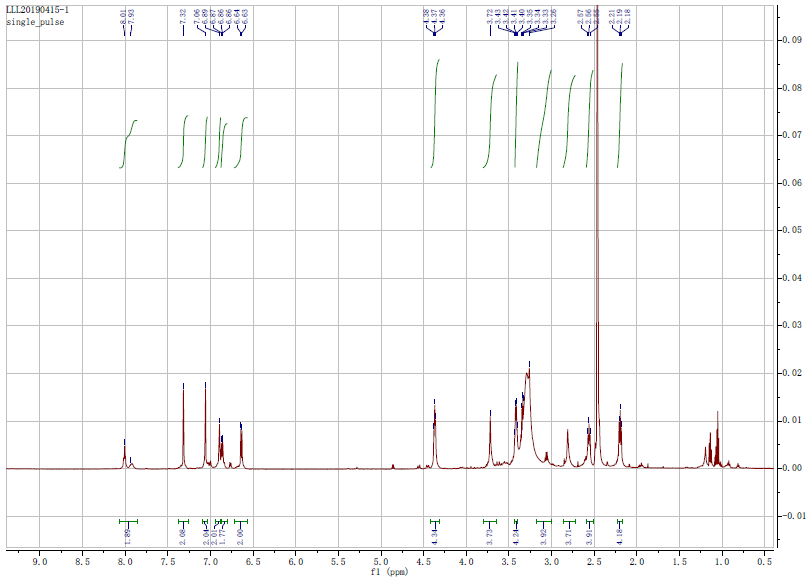


Figure S5 1H NMR of compound **L2**

Supplement: Figure S5 1H NMR of compound L2 [file rsos191247supp5.docx]

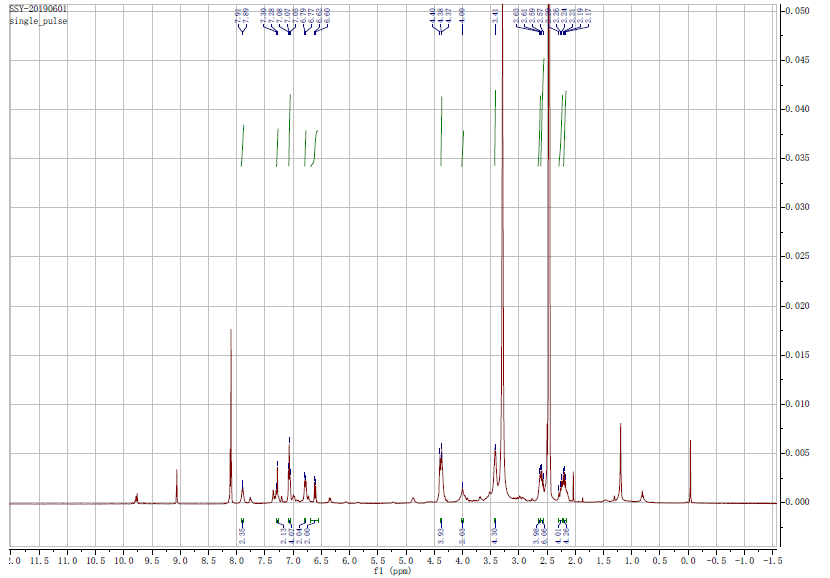


Figure S6 1H NMR of compound Re(CO)3**L2**

Supplement: Figure S6 1H NMR of compound Re(CO)3L2 [file rsos191247supp6.docx]
