# Supplementary material for: Synthesis of novel technetium-99m tricarbonyl-HBED-CC complexes and structural prediction in solution by density functional theory calculation
Source: R Soc Open Sci. 2019 Nov 27;6(11):191247. doi: 10.1098/rsos.191247 (PMC6894603; doi:10.1098/rsos.191247)
Supplement: Table S7 calculation detail about energy [file rsos191247supp7.docx]

Table S7. Computed energies of all stationary points in water at 368.15K.

| Species | E_1_  (ΔE_1_) | G_1_  (ΔG_1_) | E_2_  (ΔE_2_) | G_2_  (ΔG_2_) |
| --- | --- | --- | --- | --- |
| a1 | -6418.30691  (-18.8) | -6417.82032  (-14.4) | -6418.24579  (-27.1) | -6417.77368  (-22.9) |
| a2 | -6418.30530  (-13.5) | -6417.81547  (-7.6) | -6418.23744  (-26.1) | -6417.76291  (-19.8) |
| a3 | -6418.28153  (-9.6) | -6417.79589  (-4.3) | -6418.23119  (-11.2) | -6417.75771  (-7.5) |
| a4 | -6418.26775  (-2.3) | -6417.78250  (-1.7) | -6418.21957  (-2.5) | -6417.75345  (0.9) |
| a5 | -6418.25203  (1.1) | -6417.76755  (-0.1) | -6418.21415  (7.4) | -6417.75090  (10.2) |
| L1 | -1871.73300 | -1871.26770 | -1871.68596 | -1871.23180 |
| b1 | -7395.01914  (-25.5) | -7394.31748  (-19.1) | -7394.93378  (-33.2) | -7394.25028  (-29.9) |
| b2 | -7395.01078  (-20.6) | -7394.30943  (-15.0) | -7394.92601  (-27.9) | -7394.24368  (-24.8) |
| b3 | -7395.00906  (-20.0) | -7394.30535  (-11.8) | -7394.92504  (-26.9) | -7394.23864  (-22.3) |
| H_2_O | -76.44546 | -76.43115 | -76.44769 | -76.43309 |
| L2 | -2848.43549 | -2847.75367 | -2848.36326 | -2847.70076 |
| tc-3co3H2O | -4775.86713 | -4775.80961 | -4775.87299 | -4775.81828 |

ΔE=E +3*E_H2O_ - E _tc-3co3H2O_ -E_L_

ΔG=G +3*G_H2O_ - G _tc-3co3H2O_ -G_L_

Electronic energies calculated by B3LYP+IDSCRF/DGDZVP method at PCM model (E_1_, in Hartree; ΔE_1_, in kcal/mol).

Gibbs free energies with thermal correction calculated by B3LYP+IDSCRF/DGDZVP method at PCM model (G_1_, in Hartree; ΔG_1_, in kcal/mol).

Dispersion corrected Electronic energies calculated by B3LYP-D3+IDSCRF/DGDZVP method at SMD solvent model (E_2_, in Hartree; ΔE_2_, in kcal/mol).

Dispersion corrected Gibbs free energies with thermal correction calculated by B3LYP-D3+IDSCRF/DGDZVP method at SMD solvent model (G_2_, in Hartree; ΔG_2_, in kcal/mol).
