# Supplementary material for: Synthesis of novel technetium-99m tricarbonyl-HBED-CC complexes and structural prediction in solution by density functional theory calculation
Source: R Soc Open Sci. 2019 Nov 27;6(11):191247. doi: 10.1098/rsos.191247 (PMC6894603; doi:10.1098/rsos.191247)
Supplement: Table S8 calculation detail about coordinates [file rsos191247supp8.pdf]

Table S8. Coordinates of All optimized Stationary Points Computed at B3LYP+IDSCRF/dgdzvp level in water.

a1	N	-14.53804	7.70892	-1.90395
	C	-14.42683	7.42698	-0.42909
	C	-15.03130	6.09673	-0.00799
	C	-13.35954	8.49496	-2.37814
	C	-15.83844	8.35118	-2.26937
	C	-12.75373	4.16771	1.22865
	C	-12.56159	5.07540	2.28959
	C	-12.31998	4.60554	3.58680
	C	-12.25177	3.23236	3.83434
	C	-12.40816	2.30090	2.79738
	C	-12.64967	2.79684	1.50896
	O	-12.61119	6.41665	1.99854
	C	-12.28330	0.81155	3.05277
	C	-10.83342	0.31631	2.92281
	C	-10.67634	-1.16865	3.17571
	C	-12.08009	7.64359	-2.47211
	O	-12.24540	6.36159	-2.57421
	O	-11.57731	-1.94318	3.45953
	C	-16.18242	9.76492	-1.79846
	C	-17.38802	10.28997	-2.31648
	C	-17.82729	11.56818	-1.95823
	C	-17.07690	12.34460	-1.07196
	C	-15.88037	11.85732	-0.53350
	C	-15.45763	10.57135	-0.91028
	O	-18.10158	9.48488	-3.17247
	C	-15.04584	12.70094	0.41121
	C	-14.03903	13.59148	-0.33535
	C	-13.19404	14.45133	0.58151
	O	-13.26040	14.47342	1.80105
	H	-14.92694	8.20931	0.14853
	H	-13.37372	7.45917	-0.15015
	H	-14.96823	6.01965	1.07846
	H	-16.09406	6.06155	-0.26724
	H	-13.58260	8.88090	-3.37861
	H	-13.14724	9.35467	-1.73950
	H	-15.89832	8.33970	-3.36077
	H	-16.63214	7.67801	-1.92993
	H	-12.18228	5.31812	4.39965
	H	-12.75738	2.08969	0.68602
	H	-12.43179	6.94646	2.80651
	H	-12.65155	0.57275	4.05599
	H	-12.91300	0.25682	2.34959
	H	-10.43382	0.52780	1.92349
	H	-10.17351	0.84404	3.62215
	H	-18.76228	11.94778	-2.36974
	H	-14.53147	10.20941	-0.47554
	H	-18.91136	9.95028	-3.47673
	H	-15.69888	13.33606	1.01872
	H	-14.50114	12.05726	1.10979
	H	-13.35701	12.98752	-0.94626
	H	-14.55147	14.25944	-1.03834
	C	-15.21580	3.70007	-0.57645
	H	-14.69192	2.87721	-1.06613
	H	-16.14021	3.86379	-1.13892
	C	-15.69601	3.22223	0.79899
	O	-15.86624	1.88547	0.77701
	H	-16.26906	1.60897	1.63921
	O	-15.99198	3.92091	1.75138
	N	-14.37628	4.92289	-0.67247
	O	-10.98205	8.21909	-2.45830
	C	-12.96452	4.64412	-0.19611
	H	-12.38568	5.54572	-0.38051
	H	-12.57537	3.88421	-0.87865
	O	-9.38907	-1.56248	3.05063
	H	-9.34694	-2.53533	3.22620
	O	-12.32719	15.21981	-0.11540
	H	-11.80413	15.75646	0.53073
	C	-16.13762	5.21525	-3.25639
	C	-14.20340	6.36178	-4.75015
	C	-13.74801	3.91888	-3.64617
	O	-17.25620	4.98783	-3.48387
	O	-14.16105	6.76692	-5.83674
	O	-13.40515	2.89031	-4.05974
	Tc	-14.27606	5.65471	-2.94708
	H	-12.06713	2.88741	4.85105
	H	-17.43636	13.33518	-0.79653
a2	N	-16.83060	7.96442	-3.53032
	C	-17.24217	7.65090	-2.11623
	C	-17.85358	6.26807	-1.92705
	C	-15.69543	8.93079	-3.55667
	C	-17.94779	8.55336	-4.36565
	C	-16.10676	4.28549	-0.08551
	C	-16.32991	5.10276	1.04060
	C	-16.55530	4.52541	2.29695

	C	-16.54625	3.13642	2.44373
	C	-16.29930	2.29384	1.34967
	C	-16.07600	2.89546	0.10392
	O	-16.30667	6.46417	0.85813
	C	-16.23261	0.78814	1.51599
	C	-14.81489	0.30461	1.86369
	C	-14.70999	-1.19645	2.03525
	C	-15.87653	10.36631	-3.04728
	O	-16.84861	10.53835	-2.13796
	O	-15.62840	-1.99384	1.92249
	C	-19.24220	7.78226	-4.45688
	C	-20.44281	8.45798	-4.18806
	C	-21.70265	7.87249	-4.36987
	C	-21.73157	6.54991	-4.84559
	C	-20.55600	5.84873	-5.10791
	C	-19.27949	6.43630	-4.91385
	C	-22.97856	8.62300	-4.04105
	C	-23.46622	8.35404	-2.60735
	C	-24.74430	9.08140	-2.24674
	O	-25.37909	9.82187	-2.98272
	H	-17.96520	8.39087	-1.76052
	H	-16.35784	7.75120	-1.48382
	H	-18.09738	6.15574	-0.86928
	H	-18.78714	6.18775	-2.47921
	H	-14.86550	8.51905	-2.97432
	H	-15.33972	9.03309	-4.58303
	H	-18.17817	9.55074	-3.97708
	H	-17.51994	8.68510	-5.36643
	H	-16.73490	5.16756	3.15885
	H	-15.86315	2.25931	-0.75572
	H	-16.44382	6.92235	1.71657
	H	-16.92118	0.46838	2.30507
	H	-16.56153	0.29402	0.59598
	H	-14.09783	0.60110	1.08847
	H	-14.45706	0.77056	2.78992
	H	-20.59340	4.82302	-5.47125
	H	-22.82480	9.70028	-4.16572
	H	-23.77204	8.34081	-4.74184
	H	-23.63907	7.28276	-2.44695
	H	-22.70260	8.64102	-1.87403
	C	-17.73099	3.91638	-2.68541
	H	-17.02616	3.12195	-2.93916
	H	-18.32887	4.10970	-3.58399
	C	-18.71054	3.36701	-1.64805

	O	-18.87920	2.04267	-1.85090
	H	-19.58317	1.72303	-1.23185
	O	-19.33585	3.99644	-0.81335
	N	-16.96273	5.15291	-2.38911
	O	-15.13617	11.26015	-3.42077
	C	-15.80901	4.87861	-1.44973
	H	-15.27297	5.82003	-1.32565
	H	-15.14534	4.19640	-1.98618
	O	-13.44815	-1.57601	2.33794
	H	-13.43888	-2.56037	2.43803
	O	-25.12799	8.81746	-0.97635
	H	-25.96341	9.31679	-0.79890
	Tc	-16.14043	5.95421	-4.48589
	C	-15.71691	4.20378	-5.21606
	C	-14.30640	6.10055	-3.88429
	O	-15.46286	3.16234	-5.66154
	O	-13.19097	6.19226	-3.55828
	O	-18.19410	5.70751	-5.14480
	H	-20.38886	9.48912	-3.83538
	O	-15.33232	7.17542	-7.21244
	C	-15.62868	6.72378	-6.18383
	H	-22.69042	6.05807	-5.01252
	H	-16.72789	2.70822	3.42883
	H	-16.85280	11.49166	-1.86137
a3	N	-15.43319	6.36545	-3.62910
	C	-14.64673	7.55450	-3.98839
	C	-13.90780	7.36611	-5.33245
	C	-14.68341	5.13786	-3.43984
	C	-16.45666	6.60936	-2.59421
	C	-10.79553	8.08290	-5.55836
	C	-9.64887	8.18332	-4.74802
	C	-8.51956	7.39727	-5.01366
	C	-8.52531	6.50642	-6.09037
	C	-9.65143	6.38449	-6.91869
	C	-10.76798	7.18559	-6.63249
	O	-9.68408	9.08832	-3.71016
	C	-9.66016	5.40512	-8.07877
	C	-9.78144	3.92855	-7.64696
	C	-11.13713	3.58065	-7.06621
	C	-13.63371	5.02520	-2.32273
	O	-12.20798	3.97873	-7.50036
	C	-17.58174	7.51296	-3.05884
	C	-18.53790	7.06792	-3.99551
	C	-19.57331	7.92085	-4.39900

	C	-19.67171	9.21199	-3.86903
	C	-18.75182	9.67791	-2.92095
	C	-17.72104	8.80446	-2.53647
	O	-18.42448	5.78404	-4.47036
	C	-18.90787	11.05313	-2.29312
	C	-17.63888	11.92036	-2.26936
	C	-17.15483	12.40690	-3.61350
	O	-17.91178	12.47707	-4.62722
	H	-13.93659	7.84133	-3.19525
	H	-15.35384	8.38132	-4.09035
	H	-13.14491	6.58719	-5.23250
	H	-14.63219	6.99734	-6.06960
	H	-15.38952	4.31802	-3.25578
	H	-14.15711	4.86074	-4.35812
	H	-16.87498	5.63308	-2.32500
	H	-16.01839	7.03432	-1.67839
	H	-7.63708	7.49103	-4.38082
	H	-11.65457	7.11651	-7.25901
	H	-8.83992	9.05339	-3.21022
	H	-10.48074	5.64112	-8.76297
	H	-9.65094	3.28144	-8.52501
	H	-9.00017	3.65789	-6.93226
	H	-20.30613	7.57067	-5.12602
	H	-16.99356	9.12663	-1.79143
	H	-19.17968	5.58556	-5.06533
	H	-19.70278	11.59871	-2.81013
	H	-19.23809	10.94081	-1.25205
	H	-17.82838	12.82824	-1.67923
	H	-16.80307	11.41897	-1.77189
	C	-14.04554	9.52579	-6.55532
	H	-14.86330	9.03988	-7.09932
	H	-13.45158	10.04615	-7.32214
	C	-14.68997	10.66913	-5.76163
	O	-15.51366	11.41221	-6.32191
	N	-13.23881	8.53778	-5.89439
	O	-12.81740	4.11620	-2.27815
	C	-11.99578	8.97767	-5.26411
	H	-12.09904	9.08762	-4.17725
	H	-11.77013	9.98701	-5.62959
	O	-11.04732	2.73744	-6.01451
	H	-11.96554	2.52530	-5.71170
	O	-15.93361	12.82664	-3.73155
	C	-16.98170	15.22591	-5.40200
	C	-14.67121	14.28467	-6.49749

	C	-17.05799	13.67154	-7.63716
	O	-17.37681	16.27744	-5.11245
	O	-13.67698	14.76901	-6.84828
	O	-17.53242	13.75895	-8.69223
	Tc	-16.31259	13.47950	-5.86364
	O	-14.26925	10.83148	-4.52222
	H	-14.75432	11.59474	-4.09363
	O	-13.70355	5.99205	-1.38868
	H	-12.99868	5.81711	-0.71465
	H	-8.73060	5.50135	-8.65198
	H	-7.63608	5.90779	-6.28547
	H	-20.48442	9.85755	-4.19858
a4	N	-12.69297	9.16096	-4.59122
	C	-11.85917	9.44518	-3.42168
	C	-12.16556	8.59536	-2.17979
	C	-12.26559	9.76906	-5.83128
	C	-14.13434	9.04084	-4.40748
	C	-10.86728	5.12369	-3.07964
	C	-10.03198	4.28224	-2.33157
	C	-9.99610	2.90331	-2.55700
	C	-10.80417	2.34155	-3.54537
	C	-11.66435	3.14681	-4.31101
	C	-11.67460	4.52363	-4.06131
	O	-9.20516	4.83618	-1.32524
	C	-12.56967	2.53790	-5.36384
	C	-13.92126	2.09788	-4.77767
	C	-14.85345	1.48752	-5.80365
	C	-12.29886	8.82264	-7.03388
	O	-12.51999	7.62474	-7.01543
	O	-14.60979	1.34292	-6.99168
	C	-14.94665	10.28221	-4.02796
	C	-16.31852	10.11256	-3.74276
	C	-17.11699	11.20431	-3.38533
	C	-16.56022	12.48609	-3.31398
	C	-15.20497	12.69381	-3.59664
	C	-14.42431	11.57823	-3.94830
	O	-16.82079	8.83273	-3.82952
	C	-14.59861	14.08384	-3.55914
	C	-14.64762	14.77662	-4.93109
	C	-14.04207	16.16450	-4.93578
	O	-13.53836	16.72775	-3.97589
	H	-11.92180	10.49272	-3.07786
	H	-10.81592	9.29337	-3.72072
	H	-11.42030	8.88278	-1.41808

	H	-13.14397	8.88484	-1.78095
	H	-12.83483	10.66564	-6.12257
	H	-11.22485	10.10363	-5.74513
	H	-14.54988	8.62847	-5.33552
	H	-14.31631	8.26241	-3.66052
	H	-9.33437	2.27767	-1.96038
	H	-12.32210	5.17058	-4.65230
	H	-8.28446	4.50796	-1.45389
	H	-12.74772	3.25985	-6.16713
	H	-12.08093	1.67194	-5.82206
	H	-13.78275	1.36347	-3.97500
	H	-14.44371	2.94492	-4.31664
	H	-18.17222	11.04903	-3.16038
	H	-13.37025	11.73712	-4.16045
	H	-17.77583	8.83465	-3.60225
	H	-15.12878	14.70418	-2.82899
	H	-13.55677	14.03194	-3.22542
	H	-14.12126	14.18355	-5.68903
	H	-15.68042	14.86284	-5.29010
	C	-12.75378	6.39676	-1.26909
	H	-13.21312	5.46763	-1.61920
	H	-13.54805	6.99545	-0.80971
	C	-11.74753	6.00330	-0.19276
	O	-11.73386	4.80365	0.25752
	O	-10.82674	6.80869	0.17786
	N	-12.19334	7.14198	-2.39952
	O	-12.02836	9.50312	-8.17105
	H	-12.03161	8.86050	-8.92328
	C	-10.89830	6.63049	-2.90109
	H	-10.06594	6.94086	-2.25486
	H	-10.73974	7.08515	-3.88355
	O	-16.02494	1.10726	-5.24691
	H	-16.59300	0.72019	-5.95867
	O	-14.11790	16.73751	-6.15852
	H	-13.70757	17.63605	-6.10239
	Tc	-9.64876	5.08420	0.97346
	C	-7.87655	5.79028	1.32029
	C	-10.11417	5.38131	2.80790
	C	-9.02695	3.30771	1.42825
	O	-6.83038	6.25122	1.52058
	O	-10.41164	5.56287	3.91517
	O	-8.68989	2.22871	1.69196
	H	-10.76232	1.26752	-3.72083
	H	-17.19372	13.32574	-3.03078

a5	N	-18.78802	2.43444	-1.13491
	C	-18.70406	3.31707	0.04152
	C	-19.70058	4.48693	-0.02674
	C	-19.93657	1.52491	-1.04209
	C	-17.54589	1.65384	-1.34656
	C	-17.97139	7.08257	2.13674
	C	-17.05513	6.41978	2.97974
	C	-16.58474	7.04740	4.14090
	C	-17.01776	8.33585	4.47076
	C	-17.91989	9.02649	3.65024
	C	-18.37460	8.37638	2.49156
	O	-16.63328	5.16655	2.60870
	C	-18.40969	10.41591	4.01035
	C	-19.72144	10.38190	4.81198
	C	-20.24500	11.75377	5.18239
	C	-20.39024	1.01298	-2.40427
	O	-20.31623	-0.15245	-2.76590
	O	-19.71482	12.81938	4.90736
	C	-16.35926	2.48423	-1.78939
	C	-16.24864	2.93956	-3.11979
	C	-15.13811	3.69654	-3.51559
	C	-14.12635	3.99718	-2.59791
	C	-14.19734	3.54692	-1.27266
	C	-15.32187	2.79349	-0.90039
	O	-17.24988	2.60478	-3.99728
	C	-13.08438	3.82575	-0.28083
	C	-12.03403	2.69998	-0.25748
	C	-10.93358	2.87396	0.76100
	O	-11.00202	3.75046	1.69190
	H	-17.69305	3.72723	0.07944
	H	-18.85287	2.76314	0.98557
	H	-19.58980	4.99795	-0.99425
	H	-20.72636	4.10538	0.00262
	H	-19.73221	0.64487	-0.41144
	H	-20.78565	2.05829	-0.60553
	H	-17.27541	1.09774	-0.43113
	H	-17.75917	0.90814	-2.11853
	H	-15.86987	6.52926	4.78009
	H	-19.06357	8.90053	1.82781
	H	-15.96098	4.84301	3.24703
	H	-17.64841	10.94117	4.59636
	H	-18.56561	11.00681	3.10142
	H	-20.51071	9.86580	4.25193
	H	-19.59859	9.81174	5.74097

	H	-15.06636	4.04760	-4.54497
	H	-15.39794	2.42979	0.12489
	H	-17.03155	2.93987	-4.89376
	H	-12.58961	4.77078	-0.52980
	H	-13.49911	3.94497	0.72514
	H	-12.51219	1.73674	-0.03469
	H	-11.56186	2.57572	-1.23844
	C	-20.75059	5.91261	1.68271
	H	-20.53093	6.47036	2.59993
	H	-21.38524	5.07369	1.99507
	C	-21.62305	6.81832	0.80584
	O	-22.70425	7.25136	1.49317
	H	-23.24645	7.82104	0.89227
	O	-21.40948	7.12263	-0.35624
	N	-19.52216	5.40594	1.10834
	C	-18.48257	6.42812	0.86853
	H	-18.84130	7.20853	0.17948
	H	-17.64931	5.92288	0.37271
	O	-21.40359	11.67454	5.87543
	H	-21.69652	12.59493	6.08999
	O	-9.89979	2.11321	0.72171
	Tc	-9.00986	3.14849	2.46163
	C	-9.60571	1.97268	3.81434
	C	-7.22860	2.38562	2.63948
	C	-8.58248	4.45476	3.84134
	O	-9.98811	1.24534	4.63535
	O	-8.36129	5.24830	4.65559
	O	-6.17709	1.90625	2.71849
	O	-20.91960	1.98523	-3.17088
	H	-21.19033	1.58292	-4.03350
	H	-16.63489	8.80716	5.37524
	H	-13.27316	4.58970	-2.92631
b1	N	-14.74961	7.83974	-2.72839
	C	-13.65202	7.80988	-3.75567
	C	-12.80801	9.07131	-3.76980
	C	-15.03253	6.43917	-2.30404
	C	-15.99557	8.53025	-3.23824
	C	-9.72260	8.39962	-3.04239
	C	-9.59949	7.66503	-4.23937
	C	-8.41263	7.71968	-4.98090
	C	-7.33638	8.48846	-4.53068
	C	-7.41344	9.20753	-3.32945
	C	-8.61259	9.13834	-2.60656
	O	-10.66997	6.89975	-4.63366

	C	-6.23046	9.99720	-2.80683
	C	-5.33367	9.16239	-1.86284
	C	-4.19537	9.99334	-1.28566
	C	-13.91361	5.87786	-1.40668
	O	-13.17976	6.75339	-0.79172
	O	-4.40238	11.03819	-0.65099
	C	-16.68602	7.91700	-4.44293
	C	-16.42010	8.38249	-5.74629
	C	-17.09332	7.82882	-6.84490
	C	-18.03872	6.82103	-6.65270
	C	-18.34501	6.34891	-5.36624
	C	-17.66085	6.91810	-4.28508
	O	-15.49204	9.38546	-5.89057
	C	-19.40467	5.28548	-5.15799
	C	-20.83377	5.87043	-5.10772
	C	-21.89368	4.77815	-5.03143
	O	-21.90490	3.81819	-5.81564
	H	-14.07384	7.65345	-4.75226
	H	-13.01679	6.94845	-3.55058
	H	-12.09389	9.00782	-4.59156
	H	-13.43470	9.94717	-3.95781
	H	-15.96126	6.42563	-1.72393
	H	-15.17066	5.77704	-3.16480
	H	-16.69166	8.55579	-2.39585
	H	-15.71585	9.56026	-3.46307
	H	-8.33298	7.15412	-5.90901
	H	-8.68707	9.67690	-1.66133
	H	-10.45544	6.42026	-5.46401
	H	-5.62500	10.35559	-3.64754
	H	-6.57633	10.88095	-2.26162
	H	-5.93305	8.80493	-1.01742
	H	-4.95267	8.28001	-2.38792
	H	-16.87219	8.19167	-7.84833
	H	-17.89887	6.57870	-3.27699
	H	-15.40783	9.63430	-6.83751
	H	-19.36382	4.55121	-5.96865
	H	-19.20863	4.74289	-4.22605
	H	-20.92862	6.57280	-4.27267
	H	-21.02664	6.43581	-6.02770
	C	-11.64819	10.69784	-2.31401
	H	-11.14410	10.80514	-1.35177
	H	-12.52585	11.35099	-2.28385
	C	-10.76052	11.31782	-3.39945
	O	-9.98538	12.27623	-2.85334

	H	-9.49295	12.73056	-3.58340
	O	-10.79703	11.08543	-4.59416
	N	-12.10629	9.29268	-2.45991
	O	-13.78575	4.64839	-1.30672
	C	-10.97248	8.32540	-2.18587
	H	-11.39563	7.32430	-2.22186
	H	-10.68794	8.50181	-1.14531
	C	-14.59557	10.61268	-1.07003
	C	-15.31894	8.41697	0.31389
	C	-12.81923	9.43824	0.66852
	O	-15.09648	11.65893	-1.17115
	O	-16.19573	8.17876	1.03654
	O	-12.19694	9.76979	1.59033
	Tc	-13.84786	8.83830	-0.87309
	N	-22.84071	4.93044	-4.06253
	C	-23.94678	3.99820	-3.89707
	H	-22.78079	5.72466	-3.42853
	C	-23.54975	2.88583	-2.91456
	H	-24.81314	4.55553	-3.52797
	H	-24.18770	3.58243	-4.87835
	N	-24.63539	1.90388	-2.68876
	H	-22.67804	2.35210	-3.29696
	H	-23.27884	3.31937	-1.95010
	C	-25.86693	1.90565	-3.28544
	C	-24.65051	0.80689	-1.85173
	C	-26.56259	0.81343	-2.78042
	H	-26.16657	2.65351	-4.00680
	N	-25.79290	0.13819	-1.88826
	N	-23.55367	0.41225	-1.02209
	H	-27.57051	0.49897	-3.02592
	O	-23.69723	-0.58837	-0.30643
	O	-22.51207	1.09432	-1.06358
	N	-2.93533	9.52436	-1.51389
	C	-1.74687	10.21503	-1.03436
	H	-2.81354	8.65277	-2.02537
	C	-1.39928	9.73734	0.38368
	H	-0.92784	10.00892	-1.72994
	H	-1.95280	11.28827	-1.04178
	N	-0.20088	10.40829	0.93834
	H	-2.24016	9.93163	1.05127
	H	-1.21598	8.66114	0.38175
	C	0.55574	11.36293	0.31456
	C	0.40214	10.22696	2.16621
	C	1.57503	11.70925	1.19386

	H	0.33496	11.72752	-0.67926
	N	1.46709	10.99389	2.34323
	N	-0.06040	9.30968	3.16211
	H	2.36523	12.43497	1.03901
	O	0.56990	9.24052	4.22599
	O	-1.07239	8.62929	2.90759
	H	-6.42339	8.51834	-5.12419
	H	-18.54633	6.40050	-7.52011
b2	N	-14.82636	1.55037	-1.54130
	C	-14.29161	1.33921	-0.14929
	C	-14.68021	-0.00763	0.43884
	C	-13.89258	2.38931	-2.34985
	C	-16.22342	2.08521	-1.54109
	C	-14.49415	-3.05925	1.40610
	C	-15.26172	-2.72624	2.54103
	C	-15.00737	-3.35039	3.76980
	C	-14.00461	-4.31626	3.87453
	C	-13.23944	-4.69088	2.75987
	C	-13.50899	-4.05015	1.54295
	O	-16.26415	-1.80167	2.38884
	C	-12.15817	-5.74712	2.87003
	C	-10.79615	-5.16272	3.31169
	C	-9.71338	-6.23280	3.35128
	C	-12.63455	1.61968	-2.78947
	O	-12.72434	0.32436	-2.79899
	O	-9.41947	-6.90464	2.35162
	C	-16.53526	3.48799	-1.01905
	C	-17.88259	3.88994	-1.16120
	C	-18.30868	5.14531	-0.71631
	C	-17.39898	6.02416	-0.12388
	C	-16.05590	5.66229	0.03440
	C	-15.65220	4.39408	-0.41604
	O	-18.74213	2.99049	-1.74597
	C	-15.05520	6.62550	0.64182
	C	-14.42724	7.56467	-0.41294
	C	-13.51686	8.61004	0.21871
	O	-13.89861	9.33851	1.14651
	H	-14.65949	2.11774	0.52503
	H	-13.20432	1.44295	-0.18242
	H	-14.35849	-0.04251	1.48024
	H	-15.76548	-0.12413	0.43460
	H	-14.41879	2.71891	-3.25200
	H	-13.56917	3.28654	-1.81837
	H	-16.58045	2.02541	-2.57266

	H	-16.83753	1.37183	-0.98302
	H	-15.60979	-3.08797	4.63917
	H	-12.94553	-4.34663	0.65775
	H	-16.75006	-1.68829	3.23527
	H	-12.46434	-6.51439	3.59102
	H	-12.02235	-6.24938	1.90729
	H	-10.47583	-4.40599	2.58663
	H	-10.89598	-4.66500	4.28211
	H	-19.35462	5.42819	-0.83296
	H	-14.60927	4.12910	-0.27452
	H	-19.64481	3.37426	-1.80127
	H	-15.54189	7.24140	1.40432
	H	-14.25536	6.06820	1.14350
	H	-13.89279	6.98102	-1.17015
	H	-15.22542	8.11048	-0.93041
	C	-12.63805	-1.29482	-0.28223
	H	-12.18455	-0.50131	-0.88319
	H	-12.36754	-2.22591	-0.78492
	C	-11.91459	-1.24806	1.06211
	O	-10.73990	-1.90323	0.95985
	H	-10.23441	-1.76473	1.80089
	O	-12.25141	-0.64120	2.06437
	N	-14.11849	-1.15943	-0.34085
	O	-11.62705	2.26329	-3.11337
	C	-14.79066	-2.46298	0.04310
	H	-14.49883	-3.18825	-0.71977
	C	-16.54571	-1.13290	-2.30555
	C	-15.22352	0.09244	-4.32739
	C	-14.27467	-2.25715	-3.34822
	O	-17.65611	-1.46012	-2.18951
	O	-15.52833	0.45016	-5.38848
	O	-13.98082	-3.27414	-3.82383
	Tc	-14.71980	-0.53651	-2.56415
	N	-12.26365	8.71131	-0.30874
	C	-11.30161	9.70226	0.15258
	H	-11.98587	8.08092	-1.05810
	C	-10.49819	9.14331	1.33649
	H	-10.64407	9.95359	-0.68490
	H	-11.85203	10.59888	0.44881
	N	-9.50786	10.11005	1.86440
	H	-11.17913	8.87510	2.14540
	H	-9.96469	8.23976	1.03464
	C	-9.27144	11.37049	1.38676
	C	-8.62998	9.96200	2.91873

	C	-8.26568	11.91922	2.17376
	H	-9.81057	11.78964	0.54836
	N	-7.87569	11.03089	3.12404
	N	-8.52727	8.78190	3.72109
	H	-7.82033	12.90378	2.08795
	O	-7.70147	8.77709	4.64421
	O	-9.27290	7.82157	3.44961
	N	-9.09358	-6.41476	4.55227
	C	-8.03961	-7.40045	4.74602
	H	-9.35196	-5.82415	5.34007
	C	-6.67513	-6.77679	4.41494
	H	-8.07537	-7.73882	5.78590
	H	-8.24632	-8.25113	4.09196
	N	-5.55104	-7.72544	4.58728
	H	-6.67387	-6.42872	3.38088
	H	-6.49276	-5.91356	5.05800
	C	-5.64495	-9.02861	4.99395
	C	-4.20215	-7.51478	4.38654
	C	-4.35217	-9.53864	5.01890
	H	-6.58812	-9.50088	5.23213
	N	-3.46364	-8.58478	4.63807
	N	-3.63764	-6.27481	3.94951
	H	-4.04040	-10.54010	5.29263
	O	-2.40948	-6.21589	3.80086
	O	-4.41028	-5.32003	3.74139
	H	-15.86393	-2.29504	-0.05568
	H	-13.83310	-4.79678	4.83708
	H	-17.74591	6.99764	0.22009
b3	N	-6.46978	8.03400	-15.09004
	C	-5.02710	7.61139	-15.14147
	C	-4.15370	8.53716	-15.97203
	C	-7.03291	7.54838	-13.79852
	C	-7.26799	7.53863	-16.27394
	C	-2.54343	10.76431	-17.39972
	C	-2.33808	9.92427	-18.51413
	C	-1.08300	9.87292	-19.13857
	C	-0.03415	10.66774	-18.67647
	C	-0.21228	11.54374	-17.59395
	C	-1.47266	11.57561	-16.98509
	O	-3.40062	9.18482	-18.97006
	C	0.90963	12.45035	-17.12893
	C	1.00085	13.75202	-17.95826
	C	2.18727	14.60815	-17.53491
	C	-6.46885	8.34928	-12.60972

	O	-5.99297	9.52424	-12.88695
	O	3.34354	14.16135	-17.51299
	C	-7.39037	6.03589	-16.45234
	C	-6.50681	5.32792	-17.29172
	C	-6.66093	3.94686	-17.47641
	C	-7.69905	3.26598	-16.83880
	C	-8.61057	3.94265	-16.01300
	C	-8.43533	5.32208	-15.84393
	O	-5.50900	6.03825	-17.91303
	C	-9.77012	3.21297	-15.36482
	C	-11.01449	3.15268	-16.28065
	C	-12.13981	2.33909	-15.65543
	O	-11.97351	1.16795	-15.28416
	H	-4.94778	6.59997	-15.55132
	H	-4.65724	7.57233	-14.12151
	H	-3.12245	8.16291	-15.94716
	H	-4.47292	8.50666	-17.01290
	H	-8.12016	7.67819	-13.81061
	H	-6.82850	6.48389	-13.64374
	H	-8.26655	7.96997	-16.16585
	H	-6.81598	7.97826	-17.16437
	H	-0.93651	9.21076	-19.99154
	H	-1.63465	12.26700	-16.15827
	H	-3.13834	8.66704	-19.76306
	H	0.76470	12.70944	-16.07389
	H	1.86936	11.92883	-17.20076
	H	1.14780	13.49880	-19.01484
	H	0.06255	14.31228	-17.88605
	H	-5.96578	3.41048	-18.12174
	H	-9.14422	5.86851	-15.22160
	H	-4.97086	5.44204	-18.47914
	H	-9.47722	2.18883	-15.11324
	H	-10.04299	3.70811	-14.42564
	H	-11.34927	4.16539	-16.52939
	H	-10.74713	2.65844	-17.22215
	C	-3.90741	10.87328	-16.73787
	H	-4.05958	11.90020	-16.39763
	H	-4.67480	10.65067	-17.47634
	C	-3.17504	9.79295	-13.08249
	O	-2.98801	8.46278	-13.01003
	H	-2.93407	8.20954	-12.05341
	O	-3.16859	10.52276	-12.10660
	N	-4.20065	9.97722	-15.53180
	O	-6.50669	7.84863	-11.47589

	C	-3.16888	10.34366	-14.50557
	H	-3.21604	11.42580	-14.38252
	H	-2.17302	10.09337	-14.89645
	C	-7.00467	10.82798	-16.65997
	C	-8.32427	10.48339	-14.35640
	C	-6.20507	12.19333	-14.41317
	O	-7.40379	11.11886	-17.71438
	O	-9.43908	10.60202	-14.05426
	O	-6.04077	13.30345	-14.11646
	Tc	-6.46398	10.31869	-14.87126
	N	-13.34294	2.96977	-15.53730
	C	-14.51941	2.30721	-14.99253
	H	-13.42985	3.93835	-15.83796
	C	-14.56115	2.48465	-13.46723
	H	-15.40586	2.74317	-15.46287
	H	-14.46521	1.24839	-15.25773
	N	-15.73271	1.82649	-12.84436
	H	-13.65672	2.06382	-13.02526
	H	-14.59371	3.54629	-13.21491
	C	-16.70215	1.11226	-13.49458
	C	-16.09873	1.78841	-11.51443
	C	-17.60258	0.67968	-12.52806
	H	-16.69702	0.95793	-14.56481
	N	-17.21432	1.10816	-11.29925
	N	-15.36160	2.41235	-10.45877
	H	-18.49780	0.08637	-12.67508
	O	-15.79931	2.30912	-9.30477
	O	-14.31884	3.02622	-10.75500
	N	1.90211	15.89289	-17.17805
	C	2.92976	16.82853	-16.74422
	H	0.93973	16.22109	-17.22540
	C	3.51866	17.56092	-17.95944
	H	2.47486	17.53419	-16.04266
	H	3.70419	16.26224	-16.22086
	N	4.58172	18.52378	-17.59002
	H	3.94176	16.83409	-18.65450
	H	2.73269	18.10711	-18.48467
	C	5.03862	18.78144	-16.32607
	C	5.32358	19.35060	-18.40875
	C	6.03058	19.74797	-16.44388
	H	4.64860	18.28247	-15.44958
	N	6.19754	20.09320	-17.74667
	N	5.17991	19.41422	-19.83091
	H	6.61667	20.19579	-15.64952

	O	5.90733	20.19955	-20.45392
	O	4.33146	18.67696	-20.36799
	H	0.93323	10.60849	-19.17400
	H	-7.80027	2.19228	-16.99278
h2o	O	0.44405	-0.52250	0.00000
	H	1.41763	-0.47734	0.00000
	H	0.16163	0.41031	0.00000
L1	N	-14.36835	8.22593	-7.75543
	C	-13.29063	8.47762	-6.80204
	C	-13.46015	7.82696	-5.41894
	C	-14.09184	8.66093	-9.12063
	C	-15.73708	8.40775	-7.29265
	C	-12.37991	4.17320	-5.75829
	C	-12.82365	3.54276	-6.93930
	C	-12.87653	2.14366	-7.01619
	C	-12.48784	1.36167	-5.92492
	C	-12.03205	1.95476	-4.73918
	C	-11.98604	3.35656	-4.68869
	O	-13.17336	4.33795	-8.00093
	C	-11.62879	1.11375	-3.54329
	C	-12.81256	0.83379	-2.60279
	C	-12.44839	-0.00980	-1.39910
	C	-14.80854	7.88842	-10.26887
	O	-15.35113	6.77171	-10.02940
	O	-11.34209	-0.46659	-1.15456
	C	-16.22808	9.79044	-6.84775
	C	-17.48540	9.87588	-6.20997
	C	-17.99500	11.10711	-5.78385
	C	-17.25999	12.27997	-5.98997
	C	-16.01138	12.23644	-6.62124
	C	-15.52198	10.98494	-7.03656
	O	-18.17889	8.69816	-6.02550
	C	-15.22049	13.50428	-6.88365
	C	-15.51050	14.09387	-8.27387
	C	-14.74181	15.36394	-8.57170
	O	-13.96452	15.92424	-7.81370
	H	-13.12014	9.55160	-6.59634
	H	-12.36182	8.11788	-7.26148
	H	-12.60788	8.16321	-4.80508
	H	-14.35354	8.24012	-4.93926
	H	-14.27998	9.73207	-9.29878
	H	-13.01869	8.51976	-9.30361
	H	-16.38958	8.05018	-8.09707
	H	-15.91912	7.70991	-6.46793

	H	-13.21342	1.66971	-7.93812
	H	-11.61892	3.83954	-3.78256
	H	-13.42600	3.77833	-8.76678
	H	-11.20999	0.15962	-3.88000
	H	-10.83828	1.61868	-2.97803
	H	-13.25087	1.76898	-2.23371
	H	-13.62219	0.31906	-3.13462
	H	-18.96499	11.14569	-5.28765
	H	-14.55184	10.94853	-7.52309
	H	-19.02758	8.88326	-5.56910
	H	-15.45521	14.25658	-6.12337
	H	-14.14685	13.30326	-6.80160
	H	-15.27625	13.37033	-9.06413
	H	-16.57807	14.31877	-8.38831
	C	-14.34557	5.85048	-4.28641
	H	-14.52361	4.77676	-4.41057
	H	-15.34042	6.31354	-4.28524
	C	-13.77521	6.04075	-2.87663
	O	-14.59722	5.49589	-1.94995
	H	-14.19707	5.64325	-1.05692
	O	-12.73003	6.59957	-2.58503
	N	-13.58144	6.35973	-5.40821
	O	-14.74181	8.46716	-11.39859
	C	-12.28801	5.68358	-5.64891
	H	-11.55954	5.93382	-4.86129
	H	-11.89676	6.07994	-6.58999
	O	-13.51505	-0.21422	-0.59325
	H	-13.22059	-0.76934	0.17101
	O	-15.01564	15.83380	-9.81012
	H	-14.49002	16.66067	-9.94739
	H	-17.66796	13.23089	-5.64943
	H	-12.53196	0.27632	-6.00869
L2	N	-14.88108	6.67487	-1.64831
	C	-14.64964	6.66401	-0.20569
	C	-15.65929	5.88147	0.64594
	C	-13.84694	7.38953	-2.38579
	C	-16.25099	6.82446	-2.11952
	C	-14.31291	3.04517	1.74217
	C	-13.38765	3.72122	2.56359
	C	-13.06264	3.21818	3.83043
	C	-13.65354	2.03723	4.29227
	C	-14.56662	1.33127	3.49760
	C	-14.86607	1.85361	2.22910
	O	-12.81784	4.87358	2.07470

	C	-15.19627	0.03786	3.97529
	C	-14.46241	-1.21999	3.45353
	C	-15.18430	-2.49581	3.86524
	C	-12.46710	6.67969	-2.54048
	O	-12.36215	5.44648	-2.28662
	O	-16.36834	-2.70574	3.56220
	C	-17.03579	8.11251	-1.84618
	C	-18.42794	8.11393	-2.08219
	C	-19.19607	9.26050	-1.85048
	C	-18.58495	10.43117	-1.38684
	C	-17.20565	10.47032	-1.14962
	C	-16.45940	9.30186	-1.38581
	O	-18.98787	6.94236	-2.54724
	C	-16.52504	11.74455	-0.68895
	C	-15.95697	12.56926	-1.86836
	C	-15.34440	13.88252	-1.40140
	O	-16.00262	14.72962	-0.77912
	H	-14.63699	7.68482	0.22629
	H	-13.64703	6.25870	-0.04289
	H	-15.33834	5.99207	1.69175
	H	-16.63996	6.36683	0.58090
	H	-14.20764	7.56615	-3.40668
	H	-13.62728	8.38887	-1.97141
	H	-16.24003	6.66391	-3.20651
	H	-16.83548	5.98708	-1.72475
	H	-12.34686	3.75317	4.45467
	H	-15.56365	1.31025	1.58972
	H	-12.18418	5.23379	2.73219
	H	-15.20415	0.01435	5.07130
	H	-16.23960	-0.01655	3.64778
	H	-14.44286	-1.19923	2.35781
	H	-13.42313	-1.22187	3.79919
	H	-20.27077	9.23513	-2.03180
	H	-15.38972	9.32495	-1.20028
	H	-19.95683	7.05891	-2.65100
	H	-17.23219	12.37249	-0.13792
	H	-15.70625	11.50337	-0.00019
	H	-15.23039	11.97158	-2.42889
	H	-16.77209	12.82149	-2.55653
	C	-17.13672	3.90647	0.59892
	H	-17.22141	2.90879	0.15443
	H	-17.93363	4.50979	0.13684
	C	-17.55008	3.79673	2.07457
	O	-18.50277	2.84686	2.23559

	H	-18.79331	2.86125	3.18122
	O	-17.15639	4.49427	2.99413
	N	-15.85373	4.47858	0.26576
	O	-11.54067	7.44389	-2.95992
	C	-14.70245	3.56924	0.36052
	H	-13.85353	4.07682	-0.10253
	H	-14.93131	2.70254	-0.27278
	N	-14.03052	14.07601	-1.71247
	C	-13.31399	15.29273	-1.35942
	H	-13.52443	13.34568	-2.20900
	C	-12.68605	15.14617	0.03502
	H	-12.54619	15.46996	-2.11853
	H	-14.02526	16.12225	-1.37808
	N	-11.94219	16.35482	0.45931
	H	-13.46839	14.94984	0.76977
	H	-11.99582	14.30040	0.04777
	C	-11.79624	17.51014	-0.25945
	C	-11.25109	16.57959	1.63237
	C	-11.02704	18.37260	0.51279
	H	-12.23060	17.64844	-1.24000
	N	-10.69459	17.77988	1.68852
	N	-11.13398	15.62679	2.69336
	H	-10.70955	19.37897	0.26484
	O	-10.48089	15.95004	3.69497
	O	-11.69429	14.52290	2.55441
	N	-14.45946	-3.38964	4.59696
	C	-15.02593	-4.64082	5.07965
	H	-13.48440	-3.18727	4.80752
	C	-14.86528	-5.73605	4.01435
	H	-14.51060	-4.91441	6.00517
	H	-16.08233	-4.47081	5.30240
	N	-15.44262	-7.03407	4.43402
	H	-15.35923	-5.42424	3.09292
	H	-13.80768	-5.89004	3.79136
	C	-16.06181	-7.29822	5.62545
	C	-15.48608	-8.22712	3.74197
	C	-16.44725	-8.63326	5.59389
	H	-16.18871	-6.55166	6.39735
	N	-16.08232	-9.19946	4.41470
	N	-14.94716	-8.41732	2.43032
	H	-16.96451	-9.19180	6.36560
	O	-15.05355	-9.53900	1.91579
	O	-14.39943	-7.44604	1.87497

	H	-13.39402	1.66691	5.28339
	H	-19.19504	11.31600	-1.20931
tc-3co3H2O	C	-9.51326	3.20638	1.54815
	C	-11.74949	2.82225	3.04566
	C	-10.69522	0.76192	1.62360
	O	-8.44791	3.61271	1.75204
	O	-12.03822	2.98950	4.15486
	O	-10.34059	-0.31312	1.86899
	Tc	-11.29023	2.54551	1.19226

	H	-13.94505	2.47148	0.10793
	H	-13.54120	0.96921	0.32046
	H	-11.67637	2.18460	-1.62178
	H	-10.25624	2.83802	-1.47849
	H	-12.31467	4.70203	-0.39782
	H	-12.68565	5.06469	1.08379
	O	-13.38697	1.87826	0.65660
	O	-10.90442	2.26144	-1.01948
	O	-12.03145	4.58132	0.53473
