# Supplementary material for: Synthesis of novel technetium-99m tricarbonyl-HBED-CC complexes and structural prediction in solution by density functional theory calculation
Source: R Soc Open Sci. 2019 Nov 27;6(11):191247. doi: 10.1098/rsos.191247 (PMC6894603; doi:10.1098/rsos.191247)
Supplement: Table S9 calculation detail about frequencies [file rsos191247supp9.docx]

Table S9. Frequencies of All optimized Stationary Points Computed at B3LYP+IDSCRF/dgdzvp level in water.

| a1 | 3 10 12 14 17 22 25 31 42 48 56 59 62 69 70 73 74 75 84 88 90 92 98 108 113 128 132 138 156 160 178 183 193 194 207 225 235 241 257 290 311 318 322 330 335 336 353 357 359 389 413 426 435 447 449 451 457 462 464 464 470 471 496 502 504 510 514 516 518 531 545 564 582 589 592 608 616 621 627 628 629 630 639 643 652 670 701 730 740 743 755 770 786 788 793 801 814 822 825 830 883 886 895 905 921 929 939 941 947 954 961 964 968 992 1007 1014 1031 1032 1058 1059 1062 1079 1099 1107 1129 1134 1153 1153 1167 1170 1172 1179 1184 1185 1212 1213 1242 1258 1266 1279 1283 1284 1286 1290 1306 1307 1316 1321 1330 1334 1344 1349 1351 1351 1366 1367 1369 1382 1387 1394 1404 1416 1423 1423 1429 1438 1462 1463 1463 1463 1472 1484 1493 1494 1504 1504 1509 1517 1532 1537 1635 1638 1656 1657 1675 1768 1768 1785 1952 1971 2090 3054 3054 3064 3065 3070 3078 3083 3086 3095 3097 3098 3098 3120 3121 3122 3144 3153 3156 3157 3164 3164 3167 3179 3180 3192 3211 3420 3444 3444 3528 3535 |
| --- | --- |
| a2 | 8 12 14 18 20 23 24 33 44 48 54 62 63 70 71 74 75 78 79 88 91 92 95 107 110 115 148 153 154 163 179 184 187 191 201 219 232 243 265 286 298 315 324 329 333 358 360 362 377 420 429 438 445 449 453 459 464 465 468 472 483 498 505 512 515 516 520 523 534 537 542 559 585 591 593 617 620 625 629 629 636 639 643 655 665 668 696 716 740 744 769 783 786 786 792 799 811 825 830 837 865 883 888 895 913 916 928 941 949 959 961 971 974 986 1008 1011 1031 1031 1055 1058 1059 1077 1101 1116 1132 1143 1151 1153 1166 1172 1173 1178 1184 1185 1191 1213 1244 1258 1266 1278 1282 1285 1287 1300 1307 1312 1313 1316 1331 1335 1340 1349 1350 1355 1366 1367 1375 1379 1389 1390 1399 1419 1423 1424 1430 1438 1462 1463 1463 1463 1472 1482 1496 1501 1503 1504 1511 1513 1523 1537 1591 1638 1650 1657 1767 1768 1771 1783 1940 1963 2083 3044 3052 3054 3059 3060 3065 3085 3089 3092 3094 3098 3101 3115 3122 3124 3141 3145 3147 3155 3156 3159 3160 3164 3179 3179 3191 3393 3429 3444 3446 3529 |
| a3 | 6 14 16 16 20 23 28 29 35 41 46 48 54 62 67 78 86 87 89 99 104 110 112 122 134 140 145 155 166 172 184 188 198 216 223 233 252 257 265 274 289 302 321 330 340 343 350 354 372 375 379 407 417 445 451 452 452 456 457 472 475 483 487 490 492 494 503 520 528 542 547 558 574 595 598 606 611 617 621 623 624 652 658 669 673 680 686 712 717 741 766 785 798 807 815 819 823 831 851 864 865 882 896 914 919 927 931 946 950 966 970 973 979 984 986 999 1012 1037 1042 1051 1084 1114 1130 1147 1149 1167 1171 1179 1188 1188 1196 1198 1213 1215 1222 1239 1253 1256 1263 1265 1275 1276 1277 1289 1303 1319 1321 1328 1334 1348 1361 1364 1365 1369 1371 1373 1380 1389 1391 1404 1409 1415 1417 1426 1440 1449 1461 1464 1465 1469 1472 1476 1492 1495 1498 1503 1505 1519 1528 1538 1557 1638 1638 1653 1656 1678 1757 1767 1973 1975 2102 2990 3005 3018 3034 3038 3044 3048 3049 3056 3060 3075 3085 3097 3101 3105 3114 3116 3119 3123 3134 3158 3160 3162 3176 3180 3188 3234 3422 3439 3543 3544 |
| a4 | 7 10 12 14 17 19 21 25 30 31 37 41 47 53 65 68 69 73 75 80 88 89 90 97 102 113 137 145 152 163 181 189 189 196 224 226 248 252 266 289 300 308 312 322 341 345 355 360 364 373 394 402 424 436 449 450 454 461 464 464 472 479 485 493 496 507 509 515 516 519 530 540 584 592 597 605 619 621 627 629 629 634 636 643 649 659 670 727 733 750 759 774 787 788 795 812 820 823 831 834 865 891 892 916 924 931 940 942 951 960 962 969 979 989 1014 1029 1030 1036 1058 1059 1085 1112 1120 1145 1147 1151 1153 1163 1168 1172 1182 1184 1188 1206 1210 1225 1247 1247 1256 1272 1277 1277 1284 1289 1299 1304 1310 1316 1317 1326 1339 1343 1349 1350 1353 1364 1366 1383 1391 1411 1419 1422 1424 1425 1438 1454 1458 1460 1463 1463 1474 1477 1491 1503 1504 1514 1518 1526 1527 1532 1538 1634 1636 1654 1654 1768 1769 1783 1969 1971 2099 2964 2984 3004 3031 3048 3054 3055 3063 3065 3067 3069 3087 3090 3097 3099 3100 3103 3119 3122 3125 3161 3170 3173 3177 3188 3197 3443 3444 3445 3472 3544 |
| a5 | 7 10 13 17 20 22 23 24 26 32 37 40 41 45 54 60 67 71 72 75 78 85 91 95 99 113 119 126 130 140 154 166 168 181 197 205 236 259 276 282 289 303 318 328 332 340 343 350 362 371 382 394 405 425 440 443 446 455 457 463 465 475 482 491 494 503 504 515 520 520 532 539 567 589 594 600 609 616 621 628 629 636 655 663 666 668 711 726 735 756 780 785 788 791 794 797 799 823 829 843 876 889 896 901 922 928 948 951 952 954 961 977 985 989 997 1031 1031 1048 1056 1059 1076 1111 1127 1129 1141 1144 1152 1163 1172 1172 1184 1185 1202 1212 1213 1215 1261 1262 1269 1273 1282 1283 1286 1288 1288 1292 1307 1311 1312 1330 1343 1349 1351 1357 1360 1370 1372 1377 1381 1402 1413 1416 1422 1424 1440 1453 1461 1462 1463 1464 1480 1494 1500 1502 1503 1504 1508 1511 1516 1536 1537 1638 1638 1656 1656 1765 1768 1774 1975 1987 2108 2962 2969 3002 3004 3019 3048 3052 3054 3063 3063 3093 3097 3099 3099 3104 3109 3120 3120 3121 3134 3151 3155 3161 3161 3177 3177 3437 3441 3444 3539 3545 |
| b1 | 5 5 9 10 13 16 18 21 23 28 29 32 34 37 43 43 45 49 56 60 63 65 71 74 77 79 85 86 89 94 100 103 104 106 112 121 128 140 148 148 156 165 168 179 182 184 197 199 207 220 234 236 239 241 253 257 267 295 300 303 308 312 319 323 328 330 337 343 374 380 384 386 395 406 412 429 435 446 448 449 451 454 457 461 461 467 471 472 476 479 498 505 508 516 518 531 546 557 574 578 590 591 596 613 619 622 623 624 630 643 646 652 657 657 668 673 699 709 718 727 741 747 748 753 756 756 767 772 776 776 784 787 789 795 806 815 817 821 835 836 839 839 875 875 882 909 915 922 926 929 932 933 935 941 946 956 961 962 971 982 1007 1014 1017 1022 1031 1035 1050 1051 1058 1080 1089 1094 1095 1102 1106 1107 1127 1133 1136 1137 1152 1153 1168 1170 1172 1173 1179 1180 1203 1203 1210 1212 1248 1251 1252 1261 1264 1271 1271 1277 1278 1281 1283 1285 1295 1297 1305 1314 1322 1327 1328 1329 1338 1341 1343 1348 1353 1354 1362 1366 1366 1366 1372 1379 1384 1384 1385 1390 1391 1394 1396 1401 1419 1419 1427 1439 1439 1439 1461 1464 1471 1480 1480 1481 1486 1490 1493 1493 1494 1499 1510 1510 1510 1517 1517 1521 1526 1526 1535 1537 1552 1555 1558 1559 1637 1638 1656 1658 1673 1697 1699 1785 1949 1968 2088 3051 3053 3060 3060 3066 3080 3081 3084 3090 3094 3098 3102 3103 3105 3106 3106 3123 3125 3126 3144 3145 3152 3156 3157 3161 3162 3164 3164 3168 3172 3172 3179 3179 3189 3249 3249 3282 3283 3422 3521 3523 3529 3531 |
| b2 | 4 5 7 8 13 15 18 20 21 26 29 31 33 36 42 46 49 52 54 61 63 65 66 68 78 79 84 87 91 93 97 103 104 107 115 125 128 145 145 152 157 162 174 178 182 183 193 202 203 211 231 237 239 241 242 251 266 281 299 302 303 308 316 323 331 332 344 355 371 373 379 384 387 395 408 425 437 444 447 448 450 452 457 461 461 465 470 471 474 485 492 501 503 510 518 528 543 564 579 583 590 595 597 608 616 623 623 623 635 638 646 651 652 657 657 679 693 705 717 735 737 743 748 750 756 756 762 773 776 776 782 788 790 797 806 817 822 827 830 833 839 839 875 875 885 896 915 927 928 928 932 933 939 946 953 955 962 963 972 988 1006 1012 1018 1019 1033 1035 1050 1051 1053 1078 1093 1094 1098 1106 1106 1112 1131 1133 1136 1137 1152 1152 1166 1170 1173 1178 1178 1181 1203 1203 1210 1212 1242 1252 1253 1257 1268 1271 1271 1278 1283 1284 1285 1291 1295 1297 1313 1322 1326 1328 1328 1333 1340 1342 1345 1350 1353 1354 1366 1366 1367 1367 1370 1381 1384 1385 1386 1390 1393 1394 1405 1418 1419 1419 1430 1439 1439 1444 1462 1464 1480 1480 1480 1482 1483 1487 1491 1493 1493 1496 1505 1510 1511 1514 1517 1517 1526 1526 1532 1537 1552 1553 1558 1558 1635 1637 1656 1656 1679 1698 1698 1779 1955 1970 2091 3051 3052 3058 3062 3073 3079 3080 3081 3084 3088 3092 3104 3106 3107 3107 3109 3121 3124 3126 3140 3144 3144 3145 3146 3156 3160 3164 3164 3170 3172 3173 3179 3180 3210 3249 3249 3283 3284 3420 3522 3523 3532 3532 |
| b3 | 0 4 6 7 9 11 16 19 21 24 27 29 36 40 41 43 47 48 51 58 60 65 69 76 80 83 86 89 94 97 98 103 104 107 115 128 130 142 144 147 157 161 172 180 181 184 194 201 210 217 232 237 239 240 249 257 263 288 302 304 309 312 325 326 331 333 337 361 373 378 381 385 395 406 415 419 439 445 446 447 451 454 458 459 465 468 470 472 475 478 487 503 509 517 521 534 550 558 574 579 590 591 596 610 614 621 623 623 628 642 645 653 657 657 669 681 692 711 715 726 745 748 750 753 756 756 771 771 776 776 783 787 788 792 807 816 816 829 834 838 839 839 875 875 889 915 921 921 928 928 932 933 941 951 954 957 961 963 976 983 995 1015 1016 1025 1032 1038 1038 1050 1051 1066 1087 1094 1094 1105 1106 1107 1128 1133 1136 1136 1152 1152 1162 1171 1173 1178 1179 1201 1203 1203 1211 1212 1251 1251 1255 1260 1262 1271 1271 1271 1277 1284 1284 1290 1295 1299 1305 1314 1327 1328 1328 1338 1340 1342 1345 1354 1354 1360 1362 1366 1366 1369 1371 1381 1382 1384 1385 1391 1392 1393 1395 1413 1419 1419 1430 1439 1439 1456 1462 1463 1480 1480 1485 1485 1490 1492 1493 1493 1498 1502 1510 1510 1516 1517 1517 1522 1526 1526 1535 1536 1553 1553 1558 1558 1635 1637 1656 1657 1674 1698 1698 1775 1948 1966 2087 3038 3052 3053 3060 3060 3061 3065 3080 3081 3089 3098 3101 3105 3105 3106 3107 3124 3124 3125 3144 3145 3154 3158 3161 3162 3164 3165 3171 3172 3173 3179 3179 3182 3209 3249 3249 3284 3284 3418 3523 3523 3528 3530 |
| h2o | 1642 3659 3764 |
| L1 | 8 10 14 18 19 20 23 25 35 36 48 54 67 70 73 73 80 82 92 102 108 138 148 170 176 181 192 202 228 253 263 285 290 310 316 329 336 337 356 359 369 373 383 422 431 450 464 466 470 491 499 505 507 515 516 520 530 578 584 594 598 626 629 629 630 636 641 658 680 695 732 735 748 780 786 787 796 811 818 820 827 831 847 888 892 898 915 926 937 938 946 954 955 968 978 988 1010 1019 1028 1035 1059 1059 1079 1109 1130 1138 1144 1153 1153 1162 1163 1173 1182 1183 1189 1206 1211 1225 1246 1258 1268 1272 1277 1283 1286 1290 1300 1306 1311 1319 1323 1327 1329 1346 1350 1351 1362 1364 1371 1373 1391 1401 1405 1415 1422 1422 1423 1435 1458 1459 1462 1463 1463 1486 1491 1503 1503 1512 1514 1520 1529 1537 1621 1634 1638 1652 1656 1768 1768 1772 2935 2986 2990 2997 3051 3053 3053 3054 3059 3062 3063 3064 3090 3095 3096 3097 3105 3107 3118 3120 3156 3158 3161 3176 3177 3208 3439 3444 3445 3545 3553 |
| L2 | 5 7 8 9 14 17 19 21 23 25 27 28 30 36 36 42 45 47 53 58 60 65 69 72 77 78 88 97 101 104 107 132 137 143 150 159 169 181 182 189 195 198 204 224 232 238 240 249 258 271 293 298 301 302 309 314 324 328 332 345 368 374 379 385 394 404 413 420 432 447 450 450 455 458 462 471 477 492 504 509 512 526 545 569 574 587 590 592 603 618 623 623 628 637 654 657 657 665 683 703 714 716 736 743 748 749 755 756 761 767 775 776 781 788 791 799 803 814 816 821 827 834 839 839 848 875 875 893 911 916 924 926 931 932 939 943 948 950 953 968 972 985 996 1013 1018 1029 1032 1033 1050 1050 1083 1094 1094 1105 1106 1109 1120 1135 1135 1137 1152 1152 1160 1165 1169 1175 1176 1178 1192 1203 1203 1203 1209 1211 1243 1251 1254 1256 1266 1270 1271 1271 1278 1283 1285 1290 1297 1301 1310 1313 1321 1327 1327 1331 1340 1340 1349 1354 1354 1360 1366 1366 1366 1368 1372 1383 1384 1385 1388 1389 1393 1400 1406 1413 1419 1419 1420 1436 1439 1439 1456 1458 1461 1474 1480 1481 1486 1486 1491 1492 1493 1494 1509 1510 1517 1517 1518 1525 1526 1526 1529 1535 1552 1552 1558 1558 1623 1634 1636 1652 1653 1696 1696 1777 2917 2963 3011 3022 3027 3042 3049 3051 3059 3060 3073 3078 3080 3080 3089 3098 3103 3104 3106 3107 3110 3123 3124 3135 3144 3144 3147 3158 3160 3172 3173 3176 3176 3211 3249 3249 3283 3284 3444 3523 3524 3544 3548 |
| tc-3co3H2O | 47 60 88 89 91 99 113 118 138 148 150 164 319 322 337 340 350 388 453 454 455 478 479 479 601 602 626 648 652 685 1632 1633 1634 1990 1990 2119 3564 3564 3571 3638 3639 3639 |
